# An Interesting Case of Weil’s Disease with Diagnostic Challenges

**DOI:** 10.7759/cureus.71204

**Published:** 2024-10-10

**Authors:** Sushma Edara, Fareeza Mustafa, FNU Jitidhar, Nabin KC, Danilo Enriquez

**Affiliations:** 1 Internal Medicine, One Brooklyn Health- Interfaith Medical Center, New York City, USA; 2 Pulmonary Medicine, One Brooklyn Health- Interfaith Medical Center, New York City, USA

**Keywords:** leptospirosis with severe clinical manifestation, severe leptospirosis, spirochete, weil's disease, zoonotic infection

## Abstract

Weil’s disease represents a rare, severe form of leptospirosis, characterized by high mortality risk. The clinical presentation ranges from a self-limited febrile illness to a more severe form of infection where patients may have multiorgan failure. Here we present a case of a young immigrant with abdominal pain, vomiting, and body aches, presenting with mixed hyperbilirubinemia, acute kidney injury (AKI), and moderate thrombocytopenia. Imaging revealed granulomatous pneumonic changes. Despite negative initial studies, the combination of the cholestatic pattern of liver injury, thrombocytopenia, pulmonary and renal involvement, along with recent travel and occupation history, raised concern for leptospirosis. A positive *Leptospira *IgM explained the diverse presentation, emphasizing the need to consider uncommon infectious etiologies and conduct a thorough, comprehensive evaluation for accurate diagnosis and successful management.

## Introduction

Leptospirosis is caused by the spirochetal bacteria *Leptospira*, a zoonotic infection transmitted through infected animals through urine, mainly rodents, or ingestion of contaminated food or water. As per the Centers for Disease Control and Prevention (CDC), there are more than one million cases reported worldwide annually, with 60,000 deaths. In the United States (US), 100-150 cases are reported annually, with the majority of the cases from Puerto Rico and Hawaii. It can present with two distinct clinical syndromes, anicteric and icteric. Here we present a case of the icteric phase of leptospirosis, which is considered a severe, potentially life-threatening form of the illness and classically known as Weil’s disease. Failure to recognize and manage appropriately elevates the risk of mortality.

## Case presentation

We present a 37-year-old male with abdominal pain, nausea, vomiting, and generalized body aches for four days. The patient emigrated to the United States (US) from Mauritania (West Africa) four months before presentation. He started working at a slaughterhouse and living in a shared group home. 

The patient’s abdominal pain was diffuse, predominantly in the right upper quadrant, acute onset, dull aching, non-radiating, and 5/10 intensity. The pain was associated with anorexia and non-projectile, non-bilious vomiting containing mostly partially digested food particles, three to four episodes per day. He received intravenous fluid, antiemetics, and analgesics in the emergency room. On examination, he was alert, oriented, in mild distress, afebrile, normotensive, with scleral icterus, congested nasal cavity, injected posterior pharyngeal wall, and generalized abdominal tenderness. The patient was admitted for the management of acute kidney injury (AKI) stage 2 with rhabdomyolysis and moderate thrombocytopenia.

**Figure 1:**
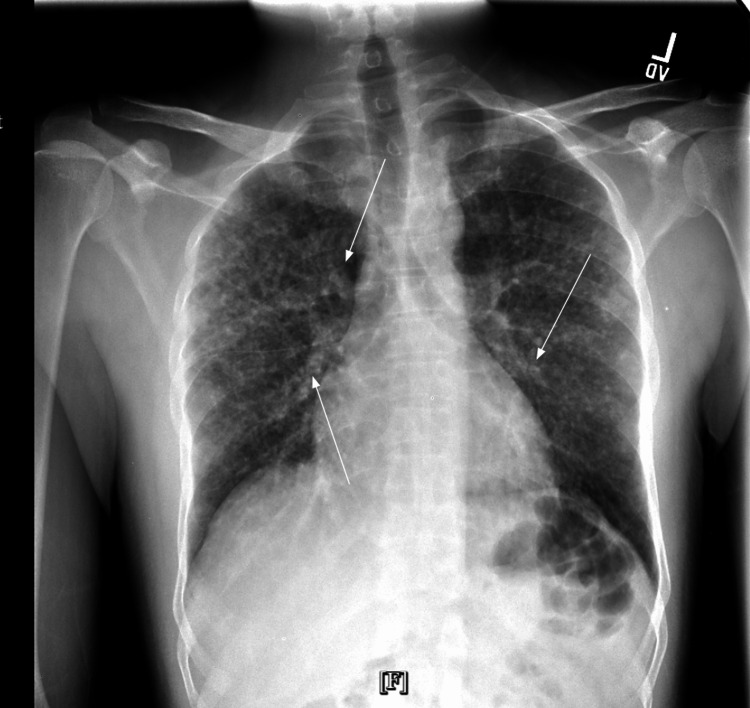
Diffuse, bilateral granular opacities (white arrows) in the chest X-ray

Chest X-rays revealed diffuse, bilateral granular opacities (Figure 1). 

**Figure 2:**
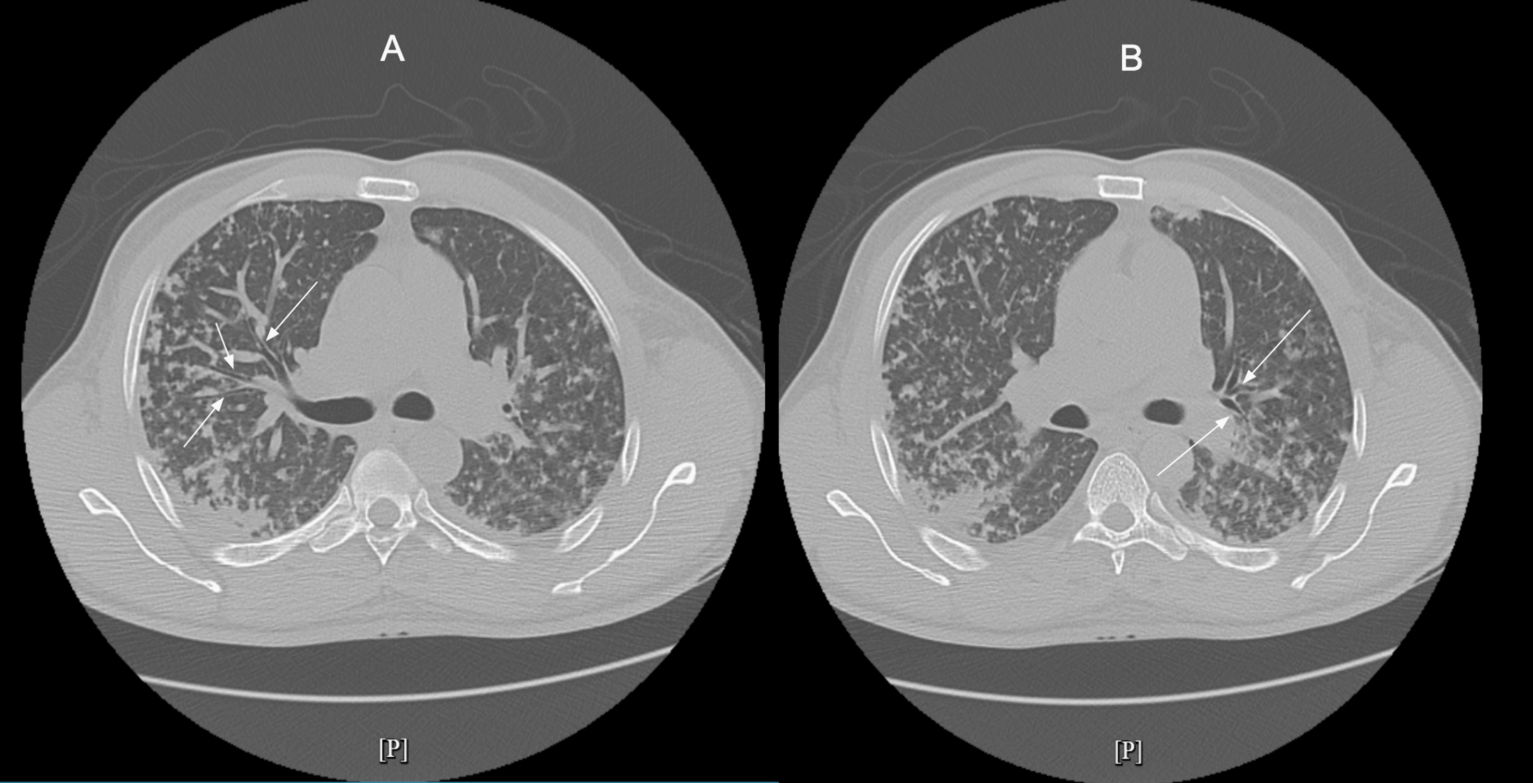
The CT scan of the chest (A and B) shows scattered bilateral multifocal opacities in a trees-in-bud pattern (white arrows). Diffuse multifocal opacities in a trees-in-bud pattern in the section of the carina (A) and below the level of the carina (B)

A computer tomography (CT) of the chest showed scattered bilateral multifocal opacities in a tree-in-bud pattern, representing an atypical infectious process (Figure 2).

Small effusions and bilateral borderline axillary adenopathy were noted. Urine microscopy indicated bacteriuria and granular casts suggestive of acute tubular necrosis (ATN). On the third day of admission, the patient reported a few episodes of hemoptysis, which prompted sputum collection to rule out tuberculosis (TB) and fungal infection. Investigations for connective tissue disease, malaria, and silicosis were sent. Along with managing the patient with ceftriaxone and azithromycin, the diagnostic plan involved bronchoscopy with transbronchial biopsy.

Iron studies suggested relative iron deficiency prompting oral iron supplementation. Elevated C-reactive protein (CRP) and procalcitonin (Table 1). Antinuclear antibody (ANA) panel, blood culture, blood parasite, mycoplasma serology, acid-fast bacteria (AFB) smear, QuantiFERON test, syphilis screening, human immunodeficiency virus (HIV) test, *Legionella *urine antigen, and urine culture all yielded negative reports. Rapid diagnostic test on admission was negative. Although the viral hepatitis panel showed no abnormalities, an elevated mitochondrial antibody was identified, associated with hyperbilirubinemia linked to primary biliary cirrhosis. A presumptive diagnosis of small duct primary biliary cholangitis (PBC) was made, supported by a negative perinuclear antineutrophil cytoplasmic antibody (pANCA). Bronchial lavage revealed *Enterococcus faecalis*, likely due to contamination. A positive *Leptospira *IgM in urine, consistent with clinical signs of leptospirosis, was managed with doxycycline, and follow-up showed resolution of symptoms. 

**Table 1:** The patient's laboratory parameters at presentation

Laboratory parameters	Day 1	Day 3	Day 5	Day 10	Day 15	Day 20	Normal reference range
Platelets	75	98	233	399	441	204	180-401 10 x 3/uL
Alanine transaminase	40	24	32	31	80	72	<35 U/L
Aspartate transaminase	49	32	30	29	61	33	14-36 U/L
Alkaline phosphatase	101	66	87	124	126	96	38-126 U/L
Total bilirubin	7.4	9.3	17	13.3	7.5	2.8	0.3-1.0 mg/dl
Direct bilirubin	4.04		8.57		4.27		0.03-0.8 mg/dl
Blood urea nitrogen	39	32	11	15	21	14	7-17 mg/dL
Creatinine	2.2	2.5	1.2	1	0.9	0.8	0.52-1.04 mg/dL
Potassium	2.9	3.2	4.4	4.6	4	3.7	3.5-5 mg/dl

## Discussion

Leptospirosis is caused by pathogenic *Leptospira interrogans*. *Leptospira interrogans* penetrates easily through broken skin, conjunctiva, and mucosa. It colonizes and multiplies in the liver, lungs, spleen, and kidneys. The incubation period ranges from two to 30 days, with most clinical features developing between five and 14 days after exposure. Leptospirosis presents as a biphasic illness: the first phase, known as the septic phase, often anicteric, lasts four to seven days. During the immune/icteric phase, less prominent fever occurs along with aseptic meningitis, uveitis, iritis, and multisystem involvement including the lungs, liver, and kidneys [1]. *Leptospira *can be found in the bloodstream and cerebral fluid during this stage. The immune phase/icteric phase lasts for four to 30 days, characterized by less prominent fever, aseptic meningitis, uveitis, iritis, pulmonary, hepatic, and renal involvement. During this phase, *Leptospira *can be found in urine and aqueous humor. A severe form of the icteric phase is called Weil’s syndrome with jaundice and renal failure. Other serious complications include pulmonary hemorrhage, rhabdomyolysis, and myocarditis. Our patient eventually progressed to develop rhabdomyolysis, jaundice, AKI, and pulmonary hemorrhage with hemoptysis, with radiological changes. 

The pathogenesis involves various mechanisms. Pulmonary involvement in leptospirosis manifests as hemorrhagic pneumonitis. Severe pulmonary hemorrhage syndrome (SPHS) can present as acute respiratory distress or massive pulmonary hemorrhage associated with hemoptysis. Radiological findings may occur with or without pulmonary symptoms, which can be diffuse, small infiltrates that may be disseminated or large areas of consolidation. These infiltrates represent interstitial and intra-alveolar hemorrhage. Pleural effusions may also be present. Computed tomography findings show extensive, bilateral, ground glass opacities [2]. Studies done by Bernardi et al. [3] and Croda et al. [4] suggest involvement on innate immune receptors and adhesion molecules along with linear deposition of IgA, IgG, and IgM and complements on the surface of alveoli, which is unique to leptospirosis pulmonary hemorrhage syndrome, not seen in other pulmonary hemorrhagic syndromes. 

In the liver, hepatocyte disorganization is due to direct damage by *Leptospira*, reflecting an elevation of liver transaminases and direct hyperbilirubinemia resulting in jaundice.

In the kidneys, *Leptospira *colonizes the renal tubules, with lymphocytic infiltration causing AKI and electrolyte imbalances such as hypokalemia, magnesium loss, and a decrease in creatinine clearance [5].

Involvement of skeletal muscles causes severe myalgia, with rhabdomyolysis. Histopathology reveals myocyte necrosis and edema [6, 7]. The glycolipoprotein (GLP) fraction of the outer envelope is implicated in the cytotoxic effects on host cells.

Due to the protean manifestations, leptospirosis can easily be misdiagnosed. Diagnosis requires a high level of suspicion, especially in non-endemic areas and in urbanized settings. Differential diagnoses include dengue, malaria, measles, brucellosis, enterovirus, and hantavirus. Diagnosis is made with clinical features, lab workup reflective of organ involvement, and diagnostic tests. Diagnostic tests depend on availability, especially in resource-limited settings, and cutoff values depend on seroprevalence for an area.

As per the CDC [8], the antibodies develop between three and 10 days from the onset of symptoms, and therefore negative serologic tests in the first week of illness do not rule out the disease. Serologic tests should be repeated after seven to 14 days if the initial result is negative and clinical suspicion remains high. Serological tests have high sensitivity and specificity; IgM-based commercial assays like IgM enzyme-linked immunosorbent assay (ELISA), immunodot assay, and lateral flow tests, which are easily available, should be confirmed with confirmatory methods such as microscopic agglutination tests (MAT) and polymerase chain reaction (PCR) tests, available at CDC and some commercial labs. Recommended samples are whole blood collected in the first week of illness (first four days is ideal), urine collected (at least one week after symptom onset is ideal), and cerebrospinal fluid from a patient with signs of meningitis.

Most cases are self-limited; the treatment approach varies as per clinical presentation. When a diagnosis is confirmed, it is important to manage patients with antibiotics, as it can shorten the length of stay and time of illness and decrease the release of organisms into urine. 

Treatment involves antibiotics and supportive care. Mild disease is treated with doxycycline 100 mg two times a day (BID) for seven days. Other options include azithromycin 500 mg once for three days. For severe disease, IV penicillin 1.5 million U every six hours is the drug of choice, and ceftriaxone 1 g to 2 g IV once daily for seven days can be given. The role of corticosteroids and plasmapheresis in severe leptospirosis remains inconclusive due to a lack of strong evidence. In our case, the patient was started on ceftriaxone and azithromycin and transitioned to doxycycline. Table 2 covers the various aspects of leptospirosis.

**Table 2:** Various aspects of leptospirosis SPHS: severe pulmonary hemorrhage syndrome; CT: computed tomography; AKI: acute kidney injury; GLP: glycoprotein; ELISA: enzyme-linked immunosorbent assay; MAT: microscopic agglutination test; PCR: polymerase chain reaction; BID: two times a day; CDC: Centers for Disease Control and Prevention

Aspect	Details
Causative agent	Pathogenic *Leptospira interrogans*
Transmission	Penetrates through broken skin, conjunctiva, and mucosa
Colonization sites	Liver, lungs, spleen, kidneys
Incubation period	2-30 days (most clinical features develop between 5-14 days after exposure)
Phases of illness	Septic/anicteric phase: 4-7 days; Immune/icteric phase: 4-30 days
Septic/anicteric phase	Flu-like illness, headache, abdominal pain, anorexia, nausea, vomiting, myalgia, conjunctival injection without discharge, maculopapular skin rash, and pharyngeal congestion. Leptospira is present in the bloodstream and cerebrospinal fluid.
Immune/icteric phase	Less prominent fever, aseptic meningitis, uveitis, iritis, pulmonary, hepatic, and renal involvement. In this phase, *Leptospira *is found in urine and aqueous humor. Severe form: Weil’s syndrome (jaundice and renal failure). Other complications: pulmonary hemorrhage, rhabdomyolysis, myocarditis.
Pathogenesis	Pulmonary: hemorrhagic pneumonitis, SPHS (acute respiratory distress, massive pulmonary hemorrhage with hemoptysis), radiological findings (diffuse infiltrates, consolidation, pleural effusions, CT: extensive, bilateral, ground glass opacities), unique immune responses (IgA, IgG, IgM, complements on alveoli). Liver: disorganized hepatocytes, elevated liver transaminases, direct hyperbilirubinemia. Kidneys: colonization of renal tubules, lymphocytic infiltration, AKI, electrolyte imbalances (hypokalemia, magnesium loss, decreased creatinine clearance). Skeletal muscles: severe myalgia, rhabdomyolysis (myocyte necrosis and edema), cytotoxic effects of the GLP fraction.
Diagnosis	High suspicion in non-endemic/urban areas. Differential diagnoses: dengue, malaria, measles, Brucellosis, enterovirus, hantavirus. Diagnosis: clinical features, organ involvement. Lab tests: Serological tests (IgM ELISA, immunodot, lateral flow tests), confirmatory methods (MAT, PCR). Samples: whole blood (first week, ideal: first four days), urine (ideal: at least 1 week after symptom onset), cerebrospinal fluid (meningitis signs).
Radiological findings	Pulmonary symptoms: diffuse, small infiltrates (disseminated or large areas of consolidation). These infiltrates represent interstitial and intra-alveolar hemorrhage. Pleural effusions may also be present. CT findings: extensive bilateral, ground glass opacities.
Histopathology findings	Skeletal muscles: myocyte necrosis and edema
CDC recommendations	Antibodies develop between three and 10 days from symptom onset. Negative serological tests in the first week do not rule out the disease; repeat after seven to 14 days. Serological tests have high sensitivity and specificity.
Treatment	Mild disease: doxycycline 100 mg BID for seven days or azithromycin 500 mg once for three days. Severe disease: IV penicillin 1.5 million U every six hours, ceftriaxone 1-2 g IV once daily for seven days. The role of corticosteroids and plasmapheresis remains inconclusive.
Antibiotics and supportive care	Essential for reducing the length of stay, and time of illness, and decreasing the release of organisms in urine.

## Conclusions

Leptospirosis, particularly in its severe form known as Weil's disease, can present with a diverse array of symptoms that affect multiple organs, making early diagnosis and treatment challenging. This case of a young immigrant with a complex presentation highlights the importance of maintaining a high index of suspicion for less common infectious diseases in non-endemic regions, especially in individuals with relevant occupational and travel histories. Despite initial negative investigations, a thorough diagnostic approach, including the identification of *Leptospira *IgM, led to the correct diagnosis and successful management. Early recognition, appropriate antibiotic therapy, and supportive care are essential in reducing mortality and ensuring patient recovery, particularly in severe cases of leptospirosis.
